# Comparison of the left ventricular apex versus other arterial cannulation sites for the operative management of acute type A aortic dissection

**DOI:** 10.1186/1749-8090-10-S1-A164

**Published:** 2015-12-16

**Authors:** Petar M Vukovic, Slobodan Micovic, Miodrag Peric, Predrag Milojevic, Ivan Nesic, Mladen Boricic, Bosko Djukanovic

**Affiliations:** 1 Department of Cardiac Surgery, Dedinje Cardiovascular Institute, Belgrade, Serbia

## Background/Introduction

The selection of the arterial inflow site for cardiopulmonary bypass during surgical treatment of patients with acute aortic dissection remains a very important issue. Several arterial cannulation sites, including left ventricular apex, were popularized over the years.

## Aims/Objectives

The aim of the study was to analyse the influence of transapical cannulation on the outcomes of surgical treatment of acute type A aortic dissection.

## Method

Between January 2010 and January 2015, emergent surgical aortic repair was performed in 158 consecutive patient with acute type A aortic dissection. In all patients open distal anastomosis was performed using deep hypothermic circulatory arrest. Patients were divided into two groups: transapical cannulation group and other cannulation sites group (including femoral and axillary artery cannulation). Operative variables and intrahospital outcomes were compared between groups.

## Results

The most frequent cannulation site was the transapical cannulation (103 patients, 65.2%). The other sites cannulation group (55 patients, 34.8%) included 33 patients with femoral and 22 patients with axillary artery cannulation. The mortality rate for the entire cohort was 17.7%. The mortality rate in the transapical group was 17.5% and 18.2% when other arterial cannulation sites were performed (p = 0.91). There was no difference in major intrahospital outcomes between groups: postoperative stroke rate was 7.8% in transapical group and 9.1% in other cannulation sites group (p = 0.77), myocardial infarction rate was 4.9% vs 5.5%, (transapical group vs other cannulation sites group respectively, p = 0.87), and the incidence of postoperative acute renal failure in transapical group was 9.7% vs. 9.1% in the other cannulation sites group (p = 0.9).

## Discussion/Conclusion

This study suggests that transapical cannulation can be routinely used as a fast and safe method to establish cardiopulmonary bypass in patients with type A aortic dissection. No difference in operative outcomes was found when transapical cannulation was compared to the other cannulation sites.

